# The Influence of South East Asia Forest Fires on Ambient Particulate Matter Concentrations in Singapore: An Ecological Study Using Random Forest and Vector Autoregressive Models

**DOI:** 10.3390/ijerph17249345

**Published:** 2020-12-14

**Authors:** Jayanthi Rajarethinam, Joel Aik, Jing Tian

**Affiliations:** 1 Environmental Health Institute, National Environment Agency, 11 Biopolis Way, #06-05/08, Singapore 138667, Singapore; joel_aik@nea.gov.sg; 2 Pre-Hospital & Emergency Research Centre, Duke-NUS Medical School, 8 College Road, Singapore 169857, Singapore; 3 Institute of Systems Science, National University of Singapore, 29 Heng Mui Keng Terrace, Block C, D & E, Singapore 119620, Singapore; tianjing@nus.edu.sg

**Keywords:** air quality, forest fires, random forest model, vector autoregressive model

## Abstract

Haze, due to biomass burning, is a recurring problem in Southeast Asia (SEA). Exposure to atmospheric particulate matter (PM) remains an important public health concern. In this paper, we examined the long-term seasonality of PM_2.5_ and PM_10_ in Singapore. To study the association between forest fires in SEA and air quality in Singapore, we built two machine learning models, including the random forest (RF) model and the vector autoregressive (VAR) model, using a benchmark air quality dataset containing daily PM_2.5_ and PM_10_ from 2009 to 2018. Furthermore, we incorporated weather parameters as independent variables. We observed two annual peaks, one in the middle of the year and one at the end of the year for both PM_2.5_ and PM_10_. Singapore was more affected by fires from Kalimantan compared to fires from other SEA countries. VAR models performed better than RF with Mean Absolute Percentage Error (MAPE) values being 0.8% and 6.1% lower for PM_2.5_ and PM_10,_ respectively. The situation in Singapore can be reasonably anticipated with predictive models that incorporate information on forest fires and weather variations. Public communication of anticipated air quality at the national level benefits those at higher risk of experiencing poorer health due to poorer air quality.

## 1. Introduction

Biomass burning is the burning of living and dead vegetation, and it can occur naturally or due to human activities. [1,2,3]. Haze, generated by biomass burning, causes air pollution that affects local air quality as well as the air quality of distant places. Haze can have detrimental impacts on human health [4,5,6,7,8], climate, biodiversity, tourism and agricultural production [9] as well as degrade visibility [10].

In recent decades, biomass burning has become a recurring phenomenon in mainland Southeast Asia (SEA) and the islands of Sumatra and Borneo [10,11,12,13,14]. The majority of biomass burning in Southeast Asia occur due to human initiated activities such as land clearing for oil palm plantations, other causes of deforestation, poor peatland management, and burning of agriculture waste [15,16]. Haze can be felt even in downwind locations such as Singapore [17,18].

Several studies have shown that meteorological conditions have significant influence on the formation of haze [19,20,21,22,23,24]. In 2012, Reid et al. [25] investigated relationships between fire hotspot appearance and various weather phenomena as well as climate variabilities in different timescales and found that the influence of these factors on fire events varies over different parts of the Maritime Continent. Haze was also shown to be worse in El Niňo years [26]. In addition, a study in Singapore demonstrated that haze fluctuates according to localities and seasons and is also influenced by factors such as weather parameters and the extent of burning in the neighboring regions [10]. 

Studies have also shown that forest fires in one area can affect air quality in surrounding countries. For example, a study on the 1997 Indonesia forest fires reported aerosols being transmitted from Kalimantan to other countries in SEA, including Singapore [27]. Reports have also shown the differences in air quality within a country. For instance, Singapore reported that the concentration of particulate matters in haze measured across the different regions in Singapore varied, according to seasonality as well as relative contribution from various source regions [10]. The significant variations in haze concentration across a small city like Singapore stresses the importance of a need for spatial and temporal modelling. A haze forecast system was established by the Met Office (MO) and the Meteorological Service Singapore (MSS) to predict haze in Singapore [28]. The modelling system developed could accurately reproduce the haze conditions observed in the Maritime Continent and in mainland Southeast Asia in 2013 and 2014. However, to the best of our knowledge, there is no long term study on the seasonality of air quality in Singapore, and no predictive modelling that provides daily air quality predictions. A daily prediction of air quality will be useful for nationwide planning for community activities.

Researchers have used several machine learning techniques to predict air quality. A novel spatiotemporal deep learning based air quality prediction method was proposed by researchers in Beijing, and the study showed that the proposed method outperformed models using the artificial neural network, regression moving average, and support vector regression techniques [29]. Another study explored three methods: (i) laboratory univariate linear regression, (ii) empirical multiple linear regression, and (iii) machine-learning-based calibration models using random forests (RF) and concluded that combining RF models with carefully controlled state-of-the-art multipollutant sensor packages improves the performances of prediction models of air quality sensors [29]. Another study, focusing on forecasting urban air pollution, showed that using different features in multivariate modelling with the M5P algorithm yields the best forecasting performances [30]. 

In this present study, we examined the association between forest fires in SEA and air quality in Singapore using different statistical models. Daily air quality forecasts will help the community to be better prepared for outdoor activities, and is especially useful for vulnerable individuals. 

## 2. Methods

### 2.1. Study Setting

**Figure 1:**
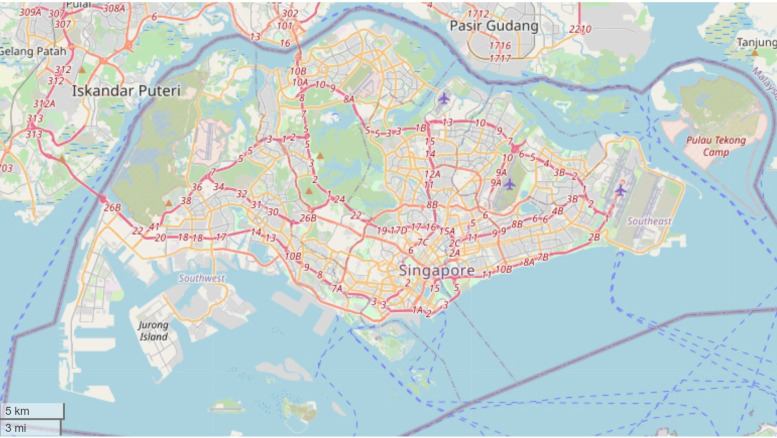
Map of study setting. Source: https://www.openstreetmap.org/#map=11/1.3680/103.8387.

We conducted our study in Singapore (1°17′ N 103°50′ E) (Figure 1), a city state with a land area of 724.2 square kilometer, and a population density of 7804 people per square kilometer, one of the highest population densities in the world [31]. Singapore experiences a tropical climate with abundant rainfall, high and uniform temperatures and high humidity all year round [32]. 

### 2.2. Climate Data

Daily mean temperature (in degrees Celsius), minimum temperature (in degrees Celsius), maximum temperature (in degrees Celsius), relative humidity (in percentage), mean wind speed (meters per speed), minimum wind speed (in meters per speed), maximum wind speed (meters per speed), wind direction (0 to 360 degrees) and total rainfall (in millimeter) from 2009 to 2018 recorded in Changi weather station in Singapore is obtained from MSS. MSS maintains a comprehensive network of specialized meteorological observing systems. It undertakes weather observation practices in accordance with international standards, and manages the long-term archive and quality control of national climate data [33]. 

### 2.3. Air Quality Data

**Figure 2:**
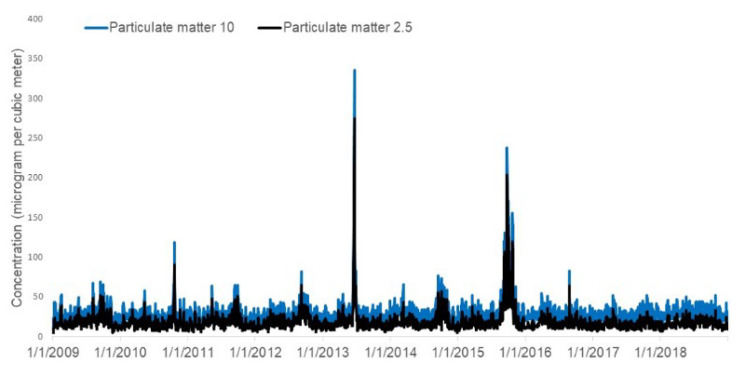
Daily distribution of particulate matter (PM_2.5_ and PM_10_ from 2009 to 2018.

Biomass burning contributes mainly to two pollutants; particulate matter 2.5 (PM_2.5_) which are particles in the air that are 2.5 micrometers or less in aerodynamic diameter, and particulate matter 10 (PM_10_), which are particles in the air that are 10 micrometers or less in aerodynamic diameter. These two pollutants are chosen for this study. The 24-h average of PM_2.5_ and PM_10_ for Singapore is recorded daily from 2009 to 2018 (Figure 2). The units for both PM_2.5_ and PM_10_ are microgram per cubic meter. Air quality readings are obtained from the USEPA AQI (United States Environmental Protection Agency Air Quality Index) system, which has been supported as an appropriate measurement by the Advisory Committee [34,35]. 

### 2.4. Forest Fire Data

**Figure 3:**
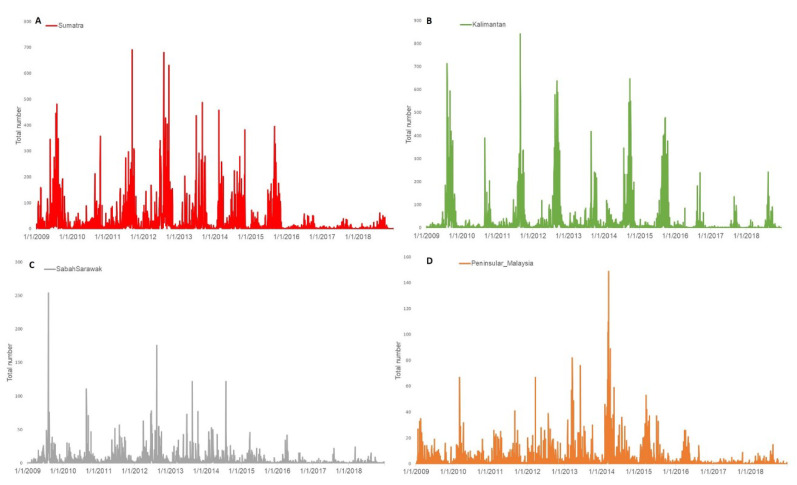
Daily distribution of forest fires hotspots counts (**A**) Sumatra (**B**) Kalimantan (**C**) Sabah/Sarawak (**D**) Peninsular Malaysia from 2009 to 2018.

Daily forest fire hotspot counts in Malaysia (Peninsular Malaysia, Sabah and Sarawak) and Indonesia (Sumatra and Kalimantan) are obtained from Association of Southeast Asian Nations Specialized Meteorological Centre for 2009 to 2018 [33] (Figure 3). The hotspots depicted are derived from the NOAA (National Oceanic and Atmospheric Administration) satellite and they represent locations with possible fires. Some hotspots may go undetected due to cloudy conditions or incomplete satellite pass. Hotspot counts from year 2016 onwards are based on the NOAA-19 satellite, and for the period from year 2006–2015 are based on the NOAA-18 satellite. The fire detection algorithm and how the hotspots are counted is described in detail on the website [36]. 

### 2.5. Statistical Analyses

The outcome variables for this study are PM_2.5_ and PM_10_. The independent variables are (i) mean temperature, (ii) minimum temperature, (iii) maximum temperature, (iv) relative humidity (v) mean wind speed, (vi) minimum wind speed, (vii) maximum wind speed, (viii) wind direction, (ix) total rainfall, (x) counts of hotspots in Kalimantan, (xi) counts of hotspots in Sumatra, (xii) counts of hotspots in Sabah and Sarawak and (xiii) counts of hotspots in Peninsular Malaysia. Each independent variable has 31 variations, with lags from 0 days to 31 days (Supplementary Table S1). Correlation tests are carried out using the “corrr” package in the R statistical language [37] to determine the association between the outcome variables and each of the independent variables. We evaluated the trend and seasonality of the daily values of PM_2.5_ and PM_10_ in separate time series models using the “ts” and “decompose” package implemented in the R statistical language [37]. The Kwiatkowski–Phillips–Schmidt–Shin (KPSS) was used to test if the time series was stationary. KPSS test for both PM_2.5_ and PM_10_ showed they were both stationary over time (*p*-value < 0.05). Therefore, the subsequent models used for prediction in this study are appropriate. 

### 2.6. Model Parameters and Evaluation

Several models such as backward stepwise multivariate regression model, Holtwinter’s Time Series model, Seasonal Autoregressive Integrated Moving Average model, RF and VAR models were explored for the analyses. We chose RF and VAR model for the following reasons. The RF model was chosen due to the ease of interpreting results; predictors that affect the outcomes most can be easily interpreted based on the importance calculation. Comparing the different time series models, VAR stands out as we can incorporate multiple independent variables into the model, which is relevant for our dataset. All the independent variables were also stationary hence the model was also appropriate for the analyses. Separate statistical models using RF and VAR techniques were built for both PM_2.5_ and PM_10_. The independent variables that were incorporated into the models can be found in Supplementary Table S1. All dataset (2009–2018) were randomly split into training (70%) dataset and testing (30%) dataset to evaluate the accuracy of the models. The accuracy of the models was tested by calculating the mean absolute percentage error (MAPE) for each model using the following equation, where *n* is the total number of fitted points:

(1) $$\frac{1}{n}\left(\sum^{\;}\frac{Actual\;value-Predicted\;value}{Actual\;value}\right)*100$$

All data and statistical analyses were performed using R software version 3.6.1 [37]. Statistical significance was assessed at the 5% level. All results, where indicated, are computed for 95% confidence intervals (CI).

### 2.7. RF Model

RF is an ensemble machine learning method that uses an ensemble of decision trees [38]. In RF, several bootstrap samples are drawn from the training set data, and an unpruned decision tree is fitted to each bootstrap sample. At each node of the decision tree, variable selection is carried out on a small random subset of the predictor variables. The best split on these predictors is used to split the node.

To find the best split for the model, we plotted the Out of Bag Error estimates and the error calculated on the test set [39]. We chose the split that gives the lowest error. We also calculated the percentage mean squared error (*MSE*) for each independent variable to determine the importance of each variable. MSE is calculated by the following equation:

(2) $$MSE\;=\;\frac{1}{n}\overset{n}{\underset{i\;=\;1}{\sum }}(Actual\;value-predicted\;value){\;}^{2}$$

Percentage *MSE* is computed by calculating the percent increase in *MSE* of the RF model when the data for each variable were randomly permuted. For each tree, the *MSE* on test is recorded. Then the same is done after permuting each predictor variable. The difference between the *MSE* on test and the *MSE* of the new model, from permuting each predictor variable, are then averaged over all trees, and normalized by the standard deviation of the differences. If the standard deviation of the differences is equal to 0 for a variable, the division is not done. The higher the difference is, the more important the variable. We categorized the top-ranked variables with a *MSE* of >10%. The predicted response is obtained by averaging the predictions of all trees. RF analyses are performed using the “Random Forest” package implemented in the R statistical language [37]. 

### 2.8. VAR Model

The VAR model extends the idea of univariate autoregression to multi time series regressions, where the lagged values of all series appear as regressors. The model can be seen as a linear prediction model that predicts the current value of a variable based on its own past value on the previous point in time and the past values of the other variables [40]. For example, the VAR model of two variables X_t_ and Y_t_ (*k* = 2) with the lag order p is defined as

Y_t_ = β_10_ + β_11_Y_t−1_ + …. + β_1p_Y_t−p_ + γ_11_X_t−1_ + …. + γ_1p_X_t−p_ + μ_1t_, (3)

X_t_ = β_20_ + β_21_Y_t−1_ + …. + β_2p_Y_t−p_ + γ_21_X_t−1_ + …. + γ_2p_X_t−p_ + μ_2t_. (4)

The βs and γs can be estimated using ordinary least squared on each equation [41]. Analyses are carried out under the assumption of normality of the data. The function “VARselect” is first used to select the maximum lag which has the lowest Akaike information criterion (AIC). The AIC is an estimator of out-of-sample prediction error and it estimates the quality of each model, relative to each of the other models. VAR analyses are conducted using the “vars” package implemented in the R statistical language [37].

## 3. Results

### 3.1. Association between PM_2.5_ and PM_10_ with Climate and Hotspots Variables 

The independent variables had weak correlation with PM_2.5_ and PM_10_; however, we noticed that for both PM_2.5_ and PM_10_ counts of hotspots in Kalimantan with lags between 1 to 18 days had an average correlation coefficient of 0.2, and *p*-value < 0.05. The correlation coefficients and corresponding *p*-values between the outcome variables (PM_2.5_ and PM_10_) and the climate and hotspot variables are listed in Supplementary Table S2. 

### 3.2. Time-Series Analyses of Daily 24-h Average of PM_2.5_ and PM_10_

**Figure 4:**
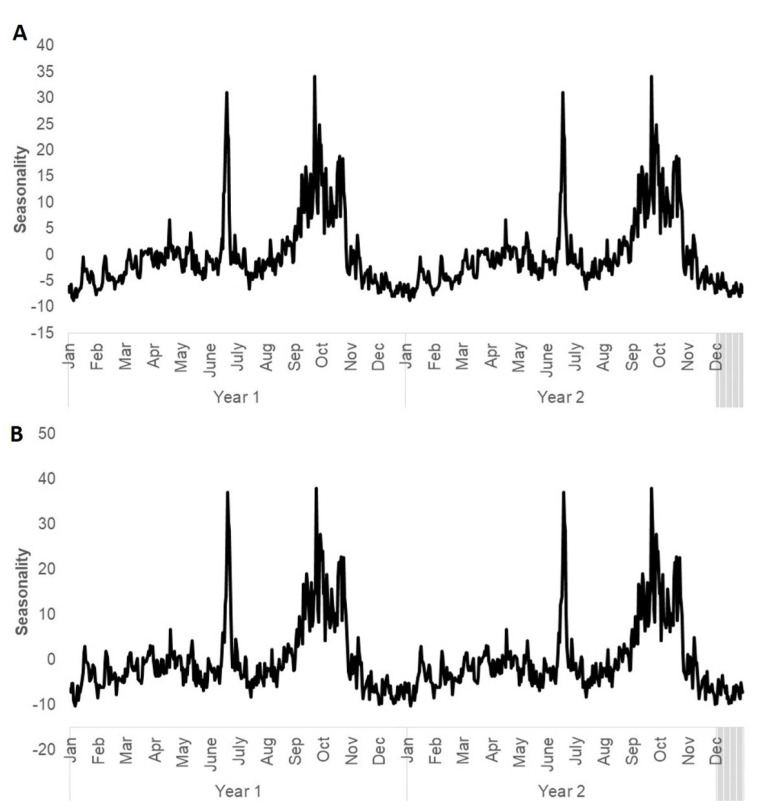
The seasonality of (**A**) PM_2.5_ and (**B**) PM_10_. The first two years are shown for easier visualization.

There are seasonal fluctuations in both PM_2.5_ and PM_10_ over the study period. We observed two annual peaks, one in the middle of the year and one at the end of the year for both PM_2.5_ (y = −2 × 10^−9^x^4^ + 3 × 10^−6^x^3^ − 0.0013x^2^ + 0.2445x − 12.161) and and PM_10_ (y = −2 × 10^−9^x^4^ + 3 × 10^−6^x^3^ − 0.0013x^2^ + 0.2316x − 11.364). There was no discerning trend, but we noticed two episodes of extremely poor air quality in mid-2013 and mid-2015, and these appeared to be outliers. Figure 4 shows the breakdown of the seasonality of PM_2.5_ and PM_10_.

### 3.3. RF Model

**Figure 5:**
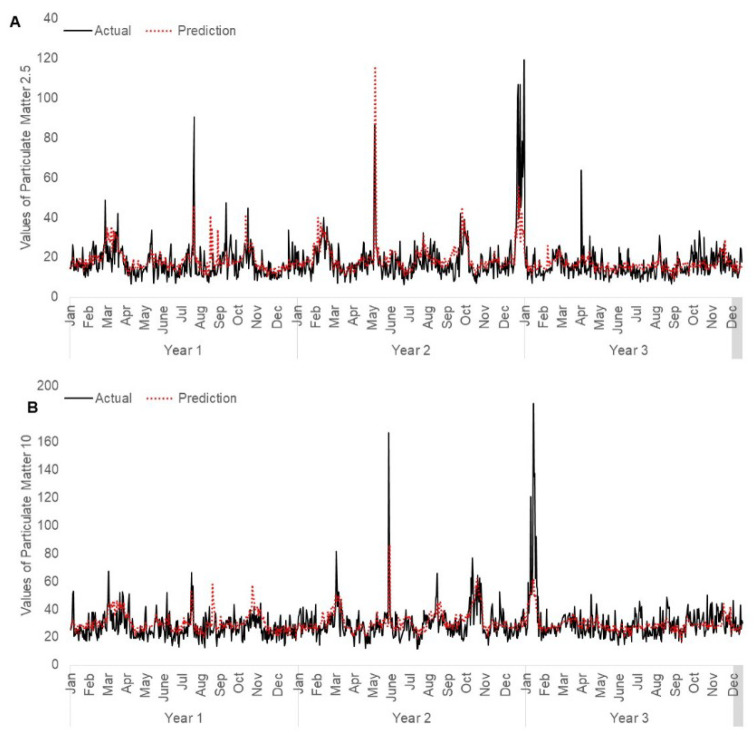
Comparison of actual and predicted air quality values using random forest model in Singapore: (**A**) PM_2.5_ and (**B**) PM_10_ Testing data (30%) is randomly selected from the dataset (2007–2018).

The RF models are built using 500 trees, and the number of variable splits that gives the lowest error for model PM_2.5_ and model PM_10_ are 193 and 89, respectively. Among the independent variables, relative humidity with lags of 0, 1 and 2 days are top-ranked for PM_2.5_ and PM_10_. In addition, counts of hotspots in Kalimantan with lags of 8 and 11 days are top-ranked for PM_2.5_, whilst counts of hotspots in Kalimantan with lags of 1, 8 and 9 days are top-ranked for PM_10_. The MSEs calculated for the rest of the variables are listed in Supplementary Table S3. Figure 5 shows graphical comparison of the predicted and actual values for PM_2.5_ and PM_10_. 

### 3.4. VAR Model

To get the lowest AIC, the VAR model for PM_2.5_ and PM_10_ was built using maximum lags of 8 and 9 respectively. The variables used in the models PM_2.5_ and PM_10_ are listed in Supplementary Table S4. Table 1 and Table 2 summarize the coefficients of the variables that were significant (*p* < 0.05) for PM_2.5_ and PM_10_, respectively. 

**Figure 6:**
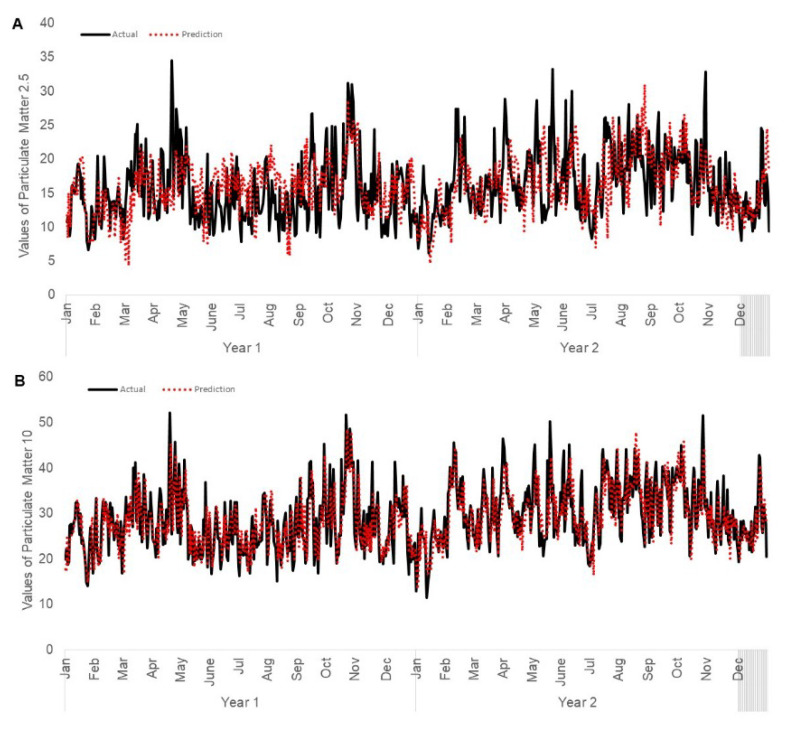
Comparison of actual and predicted air quality values using Vector Autoregressive model in Singapore: (**A**) PM_2.5_ and (**B**) PM_10_ Testing data is two years from 1 January 2017 to 31 December 2018.

Figure 6 shows the graphical comparison of the predicted and actual values for PM_2.5_ and PM_10_.

### 3.5. Comparison of Models

Table 3 shows the MAPE values for each of the four models. From Table 3, we can see that VAR models have lower MAPE performance compared to that of the RF models for both PM_2.5_ and PM_10_ experiments. 

## 4. Discussion

In this study, we sought to examine the association between forest fires and air quality in Singapore. We found a positive association between ambient air particulate concentrations in Singapore and counts of instances of forest fires. This association was observed with a 1 to 8 days’ lag depending on the location of the forest fires. Our study findings were consistent with other studies. Significant build-up of aerosol and black carbon concentrations was observed in the Tibetan plateau due to the occurrence of fires and transport of pollution from the nearby regions of Southeast Asia and the northern part of the Indian Peninsula [42]. Similarly, forest fires in Serbia resulted in air pollution through Mongolia, eastern China, down to the Korean peninsula [43]. This finding is not unexpected. Past research has shown that forest fire emissions were the largest contributors to the air pollution problem in regions tens of kilometers away from the fire source [44]. Our RF model picked up counts of hotspots in Kalimantan up to 11 days’ lag as significant variable that affects PM_2.5_ and PM_10_ concentration in the air. A similar study on the 1997 Indonesia forest fires corroborates our results that Singapore was more affected by fires from Kalimantan compared to fires from other countries, due to the shifting of the monsoons [45]. Although Malaysia and Sumatra are closer to Singapore in terms of distance than Kalimantan [46], the models show that climatic factors are important in influencing the impact of forest fires on air quality. 

Seasonality shows that the peaks in poor air quality in Singapore occurs twice, once in the middle of the year, and one at the end of the year. This finding corresponds with other studies that show that high values of PM_2.5_ and PM_10_ are reported in the middle of the year, which corresponds to the burning season [42]. Similarly, it is also reported that the burning season in SEA peaks from July to October [47]. High amounts of PM_2.5_ and PM_10_ not only aggravate health issues, but they also degrade visibility. Hence, these results can be used to guide tourism as well as large scale community programs.

- Based on our RF model’s importance plot, relative humidity is another significant variable that affects PM_2.5_ and PM_10_ concentration in the air. Other studies have also concluded that relative humidity is a key factor in influencing the distribution of air quality [48,49].
- In contrast, the VAR models picked up mean temperature lagging PM_2.5_ and PM_10_ by one and two days having significant negative effect on the concentration of PM_2.5_ and PM_10_ in the air. The effect of mean temperature on air quality has, however, been inconsistent, with several studies showing conflicting findings. Some studies have observed a negative correlation between mean temperature and concentrations of PM_2.5_ and PM_10_ [50,51]. However, there are other studies that have shown that there is a combined effect of climatic factors on the concentration of PM_2.5_ and PM_10_. For example, a study in Nagasaki, Japan concluded that temperature is positively correlated with PM_2.5_ and PM_10_ during monsoons and negatively correlated during other seasons [52]. Another study in Dhaka also showed variable response of relative humidity with air pollutants according to seasonal variation [53]. Hence, machine learning methods are relevant for the predictions of air quality, due to the mixed effects of climatic factors.
- Comparing RF and VAR models, the VAR models performs slightly better with MAPE values being 0.8% and 6.1% lower for PM_2.5_ and PM_10_, respectively. Hence, the VAR model can be reliably used for future predictions of the concentration of PM_2.5_ and PM_10_ in urban atmosphere in Singapore. To improve the communication of predictions to the community, we can categorize the predicted values according to the Table 4 [54]. Singapore uses this category to show the levels of pollutants in the air. It will be useful to release a daily prediction of PM_2.5_ and PM_10_ for community preparedness.

There are several study limitations. The fire detection algorithm used to identify forest fires hotspots is based on higher emissions of mid-infrared radiation. The fire detection algorithm compares the values of suspected fire pixels against a set of absolute thresholds, and with values of surrounding pixels. We note that hotspot detected does not always correspond to actual land fires. Other than climatic factors, there are other factors that can affect the air quality in Singapore. The models did not account for other anthropogenic sources of PM such as those from industry and shipping. Data on these factors should be collected and included into the models, to see if they can improve the predictions. In addition, currently, the dataset for independent variables were collected from Changi Meteorological Station, which is the eastern meteorological station in Singapore. Daily news reports on pollutants have shown that different parts of Singapore can be affected by the biomass burning at different intensities [55]. It will be useful to provide predictions for the five areas in Singapore (north, south, east, west and central). In order to achieve this, we need to collect climate data in different meteorological stations around the island which is spatially representative, and also obtain the measurements from the hotspots to the stations as one of the variables. The models can be further developed for better spatial resolution. Lastly, analyses were done using average values for a daily prediction. It might be more useful to the community to predict the air quality on an hourly basis. Hence, moving forward we could collect hourly data and run the models. 

## 5. Conclusions

There was a positive association between ambient air particulate concentrations in Singapore and counts of instances of forest fires, and Singapore was more affected by fires from Kalimantan compared to fires from other SEA countries. In addition, the peaks in poor air quality in Singapore occurs twice, once in the middle of the year, and one at the end of the year. VAR models performed better than RF model in predicting air quality. Our study findings suggest that air quality in Singapore can be reasonably anticipated with predictive models that incorporate information on forest fires and weather variations. The public communication of anticipated air quality at the national level benefit who are at higher risk of experiencing poorer health due to poorer air quality.

## Figures and Tables

**Table 1:** Coefficients for variables associated with PM_2.5_ that are significant (*p* < 0.05) using vector autoregressive (VAR) model.

Variables	Estimate (CI)
Mean temp with 2 days lag	−2.77 (−1.58 to −3.94)
PM_2.5_ with 1 day lag	0.76 (0.56 to 0.95)
Mean wind speed with 2 days lag	0.56 (0.10 to 1.01)
PM_2.5_ with 5 days lag	0.12 (−0.10 to 0.33)
Relative humidity with 1 day lag	−0.36 (−0.75 to 0.03)
Mean wind speed with 1 day lag	−0.44 (−0.87 to −0.01)
Mean temp with 1 day lag	−2.81 (−3.91 to −1.72)
Count of hotspots in Kalimantan with 3 days lag	0.01 (−0.08 to 0.11)
Max temp with 2 days lag	−0.63 (−1.3 to 0.04)
Count of hotspots in Kalimantan with 8 days lag	0.01 (−0.08 to 0.09)
Rainfall with 1 day lag	−0.0008 (−0.03 to 0.02)
PM_2.5_ with 6 days lag	−0.06 (−0.28 to 0.16)
Min temp with 1 day lag	0.69 (−0.05 to 1.44)
Mean wind speed with 5 days lag	0.24 (−0.21 to 0.7)
Mean wind speed with 4 days lag	−0.24 (−0.69 to 0.22)
Count of hotspots in Sabah/Sarawak with 8 days lag	−0.04 (−0.21 to 0.14)
Count of hotspots in Kalimantan with 6 days lag	0.01 (−0.08 to 0.10)
Count of hotspots in Kalimantan with 1 day lag	0.01 (−0.08 to 0.09)
Max wind speed with 2 days lag	−0.05 (−0.28 to 0.17)
Count of hotspots in Sabah/Sarawak with 6 days lag	−0.04 (−0.22 to 0.15)

**Table 2:** Coefficients for variables associated with PM_10_ that are significant (*p* < 0.05) using VAR model.

Variables	Estimate (CI)
PM_10_ with 1 day lag	0.75 (0.59 to 0.91)
Mean temp with 1 day lag	−3.53 (−2.49 to −4.56)
PM_10_ with 5 days lag	0.08 (−0.08 to 0.24)
Relative humidity with 1 day lag	−0.52 (−0.93 to −0.10)
Mean wind speed with 2 days lag	0.68 (0.19 to 1.16)
Mean temp with 2 days lag	−3.72 (−2.58 to −4.87)
Relative humidity with 2 days lag	0.31 (−0.09 to 0.72)
Mean wind speed with 1 day lag	−0.35 (−0.79 to 0.09)
Counts of hotspots in Kalimantan with 8 days lag	0.01 (−0.07 to 0.09)
Counts of hotspots in Sabah/Sarawak with 8 days lag	−0.05 (−0.24 to 0.13)
Min temp with 4 days lag	0.61 (−0.01 to 1.23)
Mean wind speed with 4 days lag	−0.33 (−0.78 to 0.13)
Rainfall with 1 day lag	−0.001 (−0.03 to 0.03)
Min temp with 1 day lag	0.84 (0.06 to 1.62)
Min temp with 2 days lag	−0.85 (−1.65 to −0.05)
Mean wind speed with 5 days lag	0.23 (−0.19 to 0.65)
Max temp with 2 days lag	−0.57 (−1.23 to 0.10)
Mean wind speed with 3 days lag	−0.23 (−0.68 to 0.22)
Counts of hotspots in Sumatra with 3 days lag	0.001 (−0.08 to 0.09)
Counts of hotspots in Sabah/Sarawak with 6 days lag	−0.04 (−0.21 to 0.14)
Rainfall with 7 days lag	0.0007 (−0.03 to 0.03)
Min temp with 9 days lag	−0.44 (−1.09 to 0.21)
Max wind speed with 2 days lag	−0.06 (−0.29 to 0.18)
Counts of hotspots in Kalimantan with 1 day lag	0.006 (−0.07 to 0.09)

**Table 3:** Mean Absolute Percentage Error of the Random Forest and Vector Autoregressive models for both PM_2.5_ and PM_10_.

	MAPE (%)	
Outcome Variable	Random Forest	VAR
PM_2.5_	26.8	26.0
PM_10_	21.3	15.2

**Table 4:** Breakdown used to define the index for PM_2.5_ and PM_10_.

Index Category	24-h PM_2.5_ (µg/m^3^)	24-h PM_10_ (µg/m^3^)
Good	0–12	0–50
Moderate	13–55	51–150
Unhealthy	56–150	151–350
Very unhealthy	151–250	351–420
Hazardous	251–350	421–500
	351–500	501–600

## Supplementary Materials

The following are available online at https://www.mdpi.com/1660-4601/17/24/9345/s1, Table S1: List of independent variables, Table S2: The correlations coefficients and corresponding *p*-values between the outcome variables (PM_2.5_ and PM_10_) and the climate and hotspot variables, Table S3: The percentage Mean Squared Errors and the corresponding increase in node purity of the variables, Table S4: The variables used in the models PM_2.5_ (Excel Sheet labelled PM_2.5_) and PM_10_ (Excel Sheet labelled PM_10_).

## List of Abbreviations

Southeast Asia: SEA, The Met Office: MO, Meteorological Service Singapore: MSS, Random forests: RF, Particulate matter 2.5: PM_2.5_, Particulate matter 10: PM_10_, USEPA: United States Environmental Protection Agency, NOAA: National Oceanic and Atmospheric Administration, Kwiatkowski–Phillips–Schmidt–Shin: KPSS, Mean Absolute Percentage Error: MAPE, Confidence Intervals: CI, Mean Squared Error: MSE, Akaike information criterion: AIC.
